# Effect of Plant Growth-Promoting Bacteria on Biometrical Parameters and Antioxidant Enzymatic Activities of *Lupinus albus* var. Orden Dorado Under Mercury Stress

**DOI:** 10.3389/fmicb.2022.891882

**Published:** 2022-06-22

**Authors:** Marina Robas Mora, Pedro Antonio Jiménez Gómez, Daniel González Reguero, Agustín Probanza Lobo

**Affiliations:** Department of Pharmaceutical and Health Sciences, School of Pharmacy, CEU Universities, Universidad CEU San Pablo, Madrid, Spain

**Keywords:** oxidative stress, bioremediation, heavy metals – contamination, plant biometry, soil bacteria

## Abstract

Heavy metal contamination of soils is a large-scale environmental problem. It leads to significant disqualification of the territory, in addition to being a source of the potential risk to human health. The exposure of plants to mercury (Hg) generates responses in its growth and their oxidative metabolism. The impact of increasing concentrations of Hg on the development of *Lupinus albus* var. Orden Dorado seedlings has been studied, as well as the plant’s response to the maximum concentration of Hg that allows its development (16 μg ml^–1^). The result shows that only the inoculum with plant growth promoting bacteria (PGPB) allows the biometric development of the seedling (root length, weight, and number of secondary roots) and prevents the toxic effects of the heavy metal from aborting the seedlings. Specifically, treatments with strains 11, 20 (*Bacillus toyonensis*), 48 (not determined), and 76 (*Pseudomonas syringae*) are interesting candidates for further PGPB-assisted phytoremediation trials as they promote root biomass development, through their PGPB activities. The plant antioxidant response has been analyzed by quantifying the catalase (CAT), superoxide dismutase (SOD), ascorbate peroxidase (APX), and glutathione reductase (GR) enzyme activity in the root, under 16 μg ml^–1^ of HgCl_2_ and different PGPB treatments. Results show that, although Hg stress generally induces enzyme activity, strains 31 and 69I (*Pseudomonas corrugata*) and 18 and 43 (*Bacillus toyonensis*) can keep SOD and APX levels close to those found in control without Hg (*p* < 0.01). Strain 18 also shows a significant reduction of GR to control levels without Hg. The present work demonstrates the benefit of PGPB treatments in situations of high Hg stress. These findings may be a good starting point to justify the role of PGPB naturally isolated from bulk soil and the rhizosphere of plants subjected to high Hg pressure in plant tolerance to such abiotic stress conditions. More studies will be needed to discover the molecular mechanisms behind the phytoprotective role of the strains with the best results, to understand the complex plant-microorganism relationships and to find effective and lasting symbioses useful in bioremediation processes.

## Introduction

The World Report on Soil Resources (FAO, 2015) identified soil contamination as one of the main threats worldwide. In this same sense, the United Nations Environmental Assembly (UNEA, 2017) adopted a resolution calling for urgent decisions to address and mitigate soil pollution. According to the World Health Organization (World Health Organization [WHO], 2007), Hg is one of the ten chemicals that pose special problems for public health. Poisoning by this heavy metal can cause death (European Commission, 2011). The organic and more labile forms of Hg, mainly methylmercury, are the most toxic for human, animal, and plant health (Peralta-Videa et al., 2009). It accumulates in biological membranes due to its high lipid solubility, which facilitates its biomagnification in the food chain (Paisio et al., 2012). In the mining district of Almadén (Spain, Ciudad Real), mining activity has led to the mobilization of mercury (Hg) into more labile forms. This mobilization has promoted an increase in the concentration of Hg in certain locations (Díaz Puente, 2013).

For Hg to be incorporated into plants with storage capacity, it must be in its bioavailable form and close to the root. Given the similarity of the Hg (II) ion with essential molecules for plants, such as zinc (Zn), copper (Cu), or iron (Fe) (Patra and Sharma, 2000), it is incorporated through the pathways by which essential micronutrients are incorporated. Once incorporated, the Hg can be retained in the roots or translocated to the aerial part of the plant (Tiodar et al., 2021) and can be recovered through harvesting. Consequently, several visible symptoms start in the root cells (Tiwari and Lata, 2018). Of all of them, the inhibition of the longitudinal growth of the root is one of the first external symptoms of the toxic effect. Measurement of the relative rate of root elongation is often used as an early indicator to differentiate between sensitive and tolerant genotypes (Sabreen and Sugiyama, 2008).

Plants are also affected by Hg by altering antioxidant enzyme systems, hindering photosynthesis, interfering with nutrient uptake, unbalancing homeostasis in general, and slowing growth (Wang et al., 2012). *Lupinus* genus (lupine) is a plant with heavy metal mobilizing capacity when grown in contaminated soils (Tounsi-Hammami et al., 2020). The choice of this species is based on its ability to adapt to various ecophysiological characteristics. This adaptability allows *Lupinus* to develop in the conditions that occur in the mines, such as high salinity, excess nitrates, and low amounts of nutrients (González et al., 2021b). The ability to solubilize and absorb soil elements thanks to powerful root development makes it behave as a candidate plant for the plant growth promoting bacteria (PGPB)-assisted phytoremediation of these ecosystems. Plant tolerance to abiotic stress depends, to a large extent, on the bacteria present in its rhizosphere to mitigate the harmful effects of pollutants (Gontia-Mishra et al., 2016; Mariano et al., 2020; González et al., 2021a). Additionally, these bacteria have been used to improve plant resistance to different abiotic stress situations, such as salinity or desiccation (Ali et al., 2022), as well as the oxidative stress produced by Hg (Ajitha et al., 2021; Çavuşoğlu et al., 2022). However, no references have been found verifying the potential of PGPB in reversing lethality resulting from exposure to high Hg concentrations.

The objective of this work is the study of the effects of PGPB inoculants of *Pseudomonas* sp. and *Bacillus* sp. genera, isolated from the rhizosphere of plants naturally grown in chronic Hg polluted soils, on the germination and development of *Lupinus albus* var. Orden Dorado, under high Hg concentrations. Once the maximum concentration of Hg tolerable by the seeds in the absence of inoculum had been determined, it was sought to demonstrate whether the incorporation of PGPB reversed necrosis and cell death, allowing plant development under conditions of high Hg stress. To this end, the study of biometric parameters (weight, length of radicles, and number of secondary roots), as well as enzymes directly related to oxidative stress in plants [superoxide dismutase (SOD), catalase (CAT), ascorbate peroxidase (APX), and glutathione reductase (GR)] were quantified as measures of the plant fitness, compared with the absence of inoculum.

## Materials and Methods

### Tested Strains

In total, twenty-four strains from bulk soil and rhizospheres of plants naturally grown in the mining district of Almadén, Ciudad Real (Spain) were used (Table 1). The experimental plot M (38°46′24.8″N, 4°51′04.7″W), is classified as an area of high contamination by Hg with concentrations of 1,710 mg kg^–1^ total Hg (Millán et al., 2011), was sampled. The plants sampled for the extraction of bacteria from the rhizosphere were *Rumex induratus* Boiss. & Reut., *Rumex bucephalophorus* L., *Avena sativa* L., *Medicago sativa* L., and *Vicia benghalensis* L., as well as bulk soil.

**TABLE 1:** Plant growth promoting bacteria (PGPB) characteristics of the tested strains.

No.	Identification 16S	Isolation origin	BMRSI (Robas et al., 2021)	MBC (μg/mL)	IAA (μg/mL)	ACCd (p/a)	Sid (cm)	PO_4_^3–^ solubility (p/a)
11	*Bacillus toyonensis*	SL	7.69	87.5	5.61 ± 0.26	+	1.0	–
18	*Bacillus toyonensis*	SL	7.87	100	6.08 ± 0.02	+	0.5	–
20	*Bacillus toyonensis*	SL	7.55	100	5.96 ± 0.12	+	0.5	–
21	*Bacillus toyonensis*	SL	7.21	100	5.31 ± 0.36	+	0.8	–
22	*Bacillus toyonensis*	SL	5.75	87.5	4.57 ± 0.08	+	0.1	–
23	*Pseudomonas moraviensis*	SL	6.97	175	4.89 ± 0.03	+	0.9	–
25	*Bacillus toyonensis*	SL	7.89	150	5.85 ± 0.11	+	0.9	–
31	*Pseudomonas corrugata*	A	7.40	100	5.60 ± 0.20	+	0.7	–
43	*Bacillus toyonensis*	A	7.68	87.5	5.70 ± 0.19	+	0.9	–
48	Nd	A	6.62	100	4.92 ± 0.24	+	0.6	–
50	*Bacillus toyonensis*	A	7.08	100	5.29 ± 0.31	+	0.7	–
57	*Pseudomonas syringae*	B	7.26	175	6.38 ± 0.30	+	0.6	–
69I	*Pseudomonas corrugata*	B	7.85	75	6.08 ± 0.08	–	0.7	–
69II	*Pseudomonas corrugata*	B	8.51	350	5.71 ± 0.13	+	0.7	+
74	*Pseudomonas syringae*	B	8.07	100	6.27 ± 0.17	+	0.7	–
76	*Pseudomonas syringae*	B	7.04	350	4.99 ± 0.05	+	0.7	–
79	*Pseudomonas syringae*	B	7.55	87.5	5.27 ± 0.31	+	0.4	–
80	*Pseudomonas syringae*	B	8.42	80	6.47 ± 0.06	+	0.8	–
112	*Pseudomonas corrugata*	C	5.61	150	4.36 ± 0.09	+	0.1	–
130	*Pseudomonas corrugata*	D	8.01	160	5.85 ± 0.12	+	1.0	–
146	*Pseudomonas fluorescens*	E	7.99	80	6.09 ± 0.11	+	0.8	–
168	*Bacillus aryabhattai*	A	6.09	87.5	6.00 ± 0.08	+	0.0	+
197	*Pseudomonas* sp.	C	5.05	80	4.97 ± 0.13	–	0.0	–
211	*Bacillus drentensis*	D	7.74	80	6.16 ± 0.02	+	0.0	–

*No, strain number; SL, bulk soil, (A–E) rhizospheres of (A) Rumex induratus, (B) Rumex bucephalophorus, (C) Avena sativa, (D) Medicago sativa, and (E) Vicia benghalensis. BMRSI, biomercury remediation suitability index; MBC, minimum bactericidal concentration; IAA, indole-3-acetic acid production; ACCd, ACC deaminase production; SID, siderophore production; Solub PO_4_^3–^, phosphate solubilization; +, positive result; –, negative result; Nd, strain not determined.*

The strains were selected based on their biomercury remediation suitability index (BMRSI) (Robas et al., 2021). This index is calculated from the variables: (i) tolerance to abiotic pressure by Hg, quantified from the calculation of the minimum bactericidal concentration (MBC), and (ii) its PGPB activities: auxin production [indole-3-acetic acid (IAA)], the presence of the enzyme 1-aminocyclopropane-1-carboxylate decarboxylase (ACCd), production of siderophores (SID), and the phosphate solubilization capacity. The BMRSI was calculated using the following formula, where the values 1 and 0 for ACCd and PO_4_^3–^ indicate presence and absence, respectively:


$$\text{BMRSI}=\left[\text{IAA}\left(\mu g.{\mathrm{mL}}^{-1}\right)+\text{ACCd}\left(1/0\right)+\text{SID}\left(\text{cm}\right)+{\mathrm{PO}}_{4}^{3\,\text{-}}\left(1/0\right)\right]+\left[\mathrm{MBC}\mathrm{Hg}\left(\mu g.{\mathrm{mL}}^{-1}\right)\right]$$

Candidates have a BMRSI > 5.5, a reference value established by the authors as a good indicator of the bioremedial capacity of the strains.

### Seed Preparation

The seeds of *L. albus* var. Orden Dorado (Seed Bank of the Center for Scientific and Technological Research of Extremadura, Spain) were sterilized following the modified protocol of Abdel Latef et al. (2017). First, a 70% (v/v) ethanol bath for 30 s, followed by two consecutive washes with sterile distilled water for 1 min each. They were imbibed in sterile water and kept refrigerated (4°C) for 24 h. Pre-germination was carried out in trays with autoclaved vermiculite and irrigated with sterile water up to field capacity. The incubation of the trays was carried out in the dark, at room temperature (20 ± 2°C), and with aluminum foil wrapping the tray superficially to avoid environmental contamination and fungal growth. The pre-germinated seeds with a visible 1 ± 0.2 cm emerged radicle, were transferred in blocks of nine seeds, to Ø140 mm Petri dishes prepared with sterile vermiculite (121°C, 1 atm, and 20 min steam heat sterilization).

### Determination of the Maximum Tolerance of Hg by the Seeds

Dilutions of Hg (using HgCl_2_) were prepared in sterile distilled water at 0.0625, 0.25, 0.5, 1.0, 2.0, 4.0, 8.0, and 16 μg.mL^–1^. Each plate, containing nine *L. albus* var. Orden Dorado seedlings, was irrigated with 50 ml of each HgCl_2_ dilution (experimental volume up to previously assayed water holding capacity, the WHO). The plates were incubated in darkness, humidity (daily hydration status check and maintenance at the WHO with distilled water), and room temperature (20 ± 2°C) for 120 h.

### Inoculation of the Seeds With the Plant Growth Promoting Bacteria Strains and Growth Conditions

A bacterial suspension was made in a sterile 0.45% saline solution (to ensure osmolarity) with a final microbial density of 0.5 McFarland. This process was repeated for each of the 24 bacterial strains tested. For each PGPB treatment, five pre-germinated *L. albus* var. Order Dorado seeds were inoculated with 1 ml of the corresponding bacterial suspension. Sterile vermiculite was brought to field capacity with 16 μg ml^–1^ HgCl_2_ dilution. The Hg-free control consisted of nine pregerminated seeds of *L. albus* var. Golden Orden watered with 50 ml of distilled water. Control with Hg was treated with 50 ml of a 16 μg ml^–1^ HgCl_2_ dilution. Both controls were without PGPB inoculum. The plates were incubated in darkness, humidity (daily hydration status check and maintenance at field capacity with distilled water), and room temperature (20 ± 2°C) for 120 h.

### Harvest

Seedlings were subjected to different PGPB treatments after 5 days of sowing (120 h), and their controls were harvested. All plants were completely removed from the substrate and washed in a 0.45% saline solution to maintain osmolarity. The root part was then excised to proceed with measurements. For each biometric and enzymatic measurement, five treatment replicates were used (*n* = 5).

### Biometry

For each treatment and control, biometric measurements of root length (cm), root weight (g), and number of secondary roots (No) were made. For root length measurement, a caliper (precision ± 0.5 mm) was used. For root weight, a precision balance (Gram RYI, FinTech, Cardiff, United Kingdom) was used (linearity and reproducibility ± 0.0002). All samples were handled minimally and under aseptic conditions. Groups of treatments with a minimum mass of 1 g were kept at −80°C for enzymatic measurements.

### Enzymatic Measurements

Catalase, SOD, APX, and GR enzyme activities were assayed.

Enzymes were extracted at 4°C from 1 g of fresh radicle sample, with a mortar and using 50 mg of polyvinylpolypyrrolidone (PVPP) and 10 ml of the following medium: 50 mM K-phosphate buffer (pH 7.8) with 0.1 mM EDTA (for SOD, CAT, and APX). The same medium, supplemented with 10 Mm of β-mercaptoethanol, was used for the GR extraction.

#### Superoxide Dismutase Activity

This activity was measured based on the ability of SOD to inhibit the reduction of nitro blue tetrazolium (NBT) by photochemically generated superoxide radicals. One unit of SOD is defined as the amount of enzyme needed to inhibit the reduction rate of NBT by 50% at 25°C (Burd et al., 2000).

#### Catalase Activity

This activity was measured following the Aebi (1984) method. H_2_O_2_ consumption was monitored for 1 min at 240 nm (Spectrophotometer visible GENESYS*™* 30, Thermo Fisher Scientific*™*, Hampton, NH, United States). This was done by mixing 50 mM potassium phosphate buffer with 10 mM H_2_O_2_ and 100 μl of the extract.

#### Ascorbate Peroxidase Activity

This activity was measured in a 1 ml reaction containing 80 nM potassium phosphate buffer, 2.5 mM H_2_O_2_, and 1 M sodium ascorbate. H_2_O_2_ was added to start the reaction and the decrease in absorbance was measured for 1 min at 290 nm, (Spectrophotometer visible GENESYS*™* 30, Thermo Fisher Scientific*™*, Hampton, NH, United States) to determine the oxidation rate of ascorbate (Amako et al., 1994).

#### Glutathione Reductase Activity

This activity was estimated spectrophotometrically at 25°C, according to the Carlberg and Mannervik (1985) method. The method is based on the measurement at 340 nm of the reduction of NADPH oxidation (Spectrophotometer visible GENESYS*™* 30, Thermo Fisher Scientific*™*, Hampton, NH, United States). The reaction mixture contained 50 mM Tris-MgCl_2_ buffer, 3 mM, 1 mM GSSG, 50 μl enzyme, and 0.3 mM NADPH, added to start the reaction. The activity was calculated with the initial rate of the reaction and the molar extinction coefficient of NADPH (ε_340_ = 6.22 mM^–1^ cm^–1^).

### Statistical Analysis

The Kruskal–Wallis statistical analysis was carried out based on non-parametric tests (*N* < 30). The variables analyzed were root length and weight, number of secondary roots, and enzymatic activities (SOD, CAT, APX, and GR). For those cases in which the value of *p* < 0.01, a *post hoc* pairwise comparison test was carried out for all treatments against controls, using the non-parametric Mann–Whitney *U*-test, applied to two independent samples. Statistically different results were those for which the value of *p* was <0.01. Duncan’s test was used to group the treatments, based on the difference in their means. A principal component analysis (PCA) was carried out for the reduction of the model. The projection of the two variables that best explain the model on the two-dimensional plane was carried out to study possible groupings between treatments. All analyses were carried out using SPSS Statistics AMOS*™* v. 27.0 software (IBM^®^ Company, Armonk, NY, United States).

## Results

**FIGURE 1:**
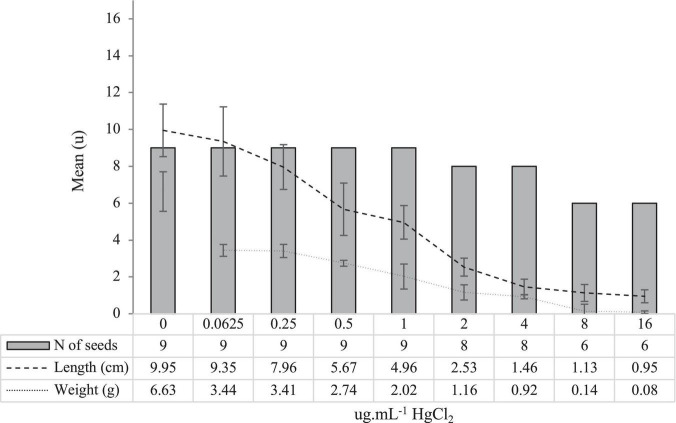
The mean (*n* = 5 of experimental units with 9 seeds) germination number of seeds (No), root length (cm), and root weight (g) for each concentration of HgCl_2_ tested (μg ml^–1^), after 120 h of development.

The results on seed germination and biometry under increasing concentrations of HgCl_2_ are shown in Figure 1.

Results show the ability of *L. albus* var. Orden Dorado seeds to tolerate high concentrations of Hg, to the detriment of root elongation and weight. The highest concentration at which the seeds were able to germinate was selected (16 μg mL^–1^).

The biometry results in the presence of the PGPB strains and 16 μg mL^–1^ of HgCl_2_ are shown in Table 2.

**TABLE 2:** The mean values of replicates (*n* = 5) ± SD for root length (cm), root weight (g) of the different treatments, and controls (absence of Hg and presence of Hg).

No.	Length (cm)	Weight (g)	Sec. roots (No)	No.	Length (cm)	Weight (g)	Sec. roots (No)
11	15.5 ± 3.87	0.78 ± 0.16	0.00	69II	9.73 ± 0.88	0.5 ± 0.06^b^	1.20
18	13.3 ± 1.10	0.61 ± 0.07	6.50	74	13.05 ± 0.91	0.70 ± 0.09	0.50
20	17.1 ± 0.61	0.81 ± 0.15	0.50	76	17.18 ± 1.05	0.79 ± 0.11	0.25
21	6.50 ± 0.76^a^	0.31 ± 0.05^b^	0.00	79	13.70 ± 0.81	0.79 ± 0.03	0.00
22	8.10 ± 0.29^a^	0.36 ± 0.05^b^	0.00	80	7.23 ± 2.75^a^	0.40 ± 0.05^b^	0.00
23	12.93 ± 0.57	0.75 ± 0.11	0.75	112	13.68 ± 1.48	0.72 ± 0.12	0.00
25	8.60 ± 0.86^a^	0.5 ± 0.08^b^	0.00	130	12.83 ± 0.99	0.68 ± 0.05	2.00
31	11.95 ± 0.47	0.70 ± 0.07	1.00	146	13.50 ± 1.41	0.74 ± 0.05	0.00
43	16.40 ± 2.15	0.65 ± 0.10	1.20	168	7.83 ± 0.30^a^	0.37 ± 0.05^b^	1.25
48	15.77 ± 1.50	0.85 ± 0.13	0.00	197	14.53 ± 0.87	0.85 ± 0.15	0.75
50	10.82 ± 1.68	0.50 ± 0.02^b^	0.00	211	13.10 ± 0.87	0.89 ± 0.06	0.75
57	7.57 ± 0.48^a^	0.27 ± 0.03	0.00	Cont. −Hg	7.63 ± 0.06^a^	0.42 ± 0.01^b^	0.15
69I	14.82 ± 1.72	0.52 ± 0.13^b^	2.00	Cont. +Hg	1.48 ± 0.02	0.05 ± 0.00	0.00

*Data with superscripts indicate that there are no significant differences with control without Hg (Duncan’s test; “a” for length and “b” for weight). The absence of superscripts means that the means are statistically different from control without Hg. The shaded cells indicate that the differences with control without Hg are statistically significant (p < 0.05). The number of secondary roots is not accompanied by ± SD since it is a discrete variable.*

Seeds treated with strains 18, 31, 43, 69I, 69II, and 130 develop secondary roots in at least three of their replicates. Specifically, the treatment with strain 20, makes the five replicates develop secondary roots in values that range between 1 and 6 secondary roots per seedling.

All biometric parameters in control without PGPB inoculum, both in the presence of Hg and in its absence, were significantly lower. The observation of the seeds in control with Hg shows that the root elongation was minimal and did not undergo variations concerning the moment of sowing (emerged radicle in pre-germination). The accumulation of Hg in embryonic tissue was evident to the naked eye.

The results of the antioxidant response in the presence of the PGPB strains and 16 μg ml^–1^ of HgCl_2_ are shown in Table 3. Treatments with strains 18, 31, 43, and 69I maintain the levels of antioxidant enzymatic activity of control without Hg (*p* < 0.01), for the enzymes SOD and APX. Strain 18, on the other hand, maintains GR levels statistically analogous to those of control without Hg (*p* < 0.01).

**TABLE 3:** The mean values of replicates (*n* = 5) ± SD for the antioxidant enzymatic activity (enzyme mg Prot^–1^ min^–1^) in the presence of PGPB treatments and controls (absence of Hg and presence of Hg).

No.	CAT	SOD	APX	GR	No	CAT	SOD	APX	GR
11	3.60 ± 0.18	8.63 ± 0.34	9.18 ± 0.45	3.78 ± 0.16	69II	7.05 ± 0.64	13.07 ± 0.42	11.02 ± 0.35	3.78 ± 0.16
18	2.23 ± 0.20	3.30 ± 0.40^a^	3.48 ± 0.66^a^	1.40 ± 0.78^a^	74	3.60 ± 0.18	8.23 ± 0.51	9.18 ± 0.71	3.56 ± 0.16
20	4.20 ± 0.16	9.36 ± 0.48	10.90 ± 0.67	3.17 ± 0.18	76	4.20 ± 0.16	9.36 ± 0.48	5.02 ± 0.42	3.37 ± 0.67
21	6.14 ± 0.39	11.58 ± 0.41	11.82 ± 0.37	4.86 ± 0.46	79	2.64 ± 0.17	6.40 ± 0.63	6.73 ± 0.68	2.56 ± 0.12
22	7.42 ± 0.40	8.42 ± 0.65	9.42 ± 0.28	10.42 ± 0.26	80	4.62 ± 0.09	11.05 ± 0.41	11.38 ± 0.39	4.41 ± 0.59
23	3.68 ± 0.51	10.70 ± 0.32	5.77 ± 0.33	3.41 ± 0.67	112	4.70 ± 0.09	6.58 ± 0.53	6.73 ± 0.70	4.66 ± 0.12
25	5.47 ± 0.33	12.79 ± 0.31	12.09 ± 0.28	5.49 ± 0.33	130	5.83 ± 0.08	13.83 ± 0.66	15.18 ± 0.21	5.95 ± 0.44
31	2.81 ± 0.41	4.99 ± 0.16^a^	3.69 ± 0.13^a^	3.27 ± 0.31	146	7.78 ± 0.08	18.27 ± 0.96	19.79 ± 0.65	7.8 ± 0.50
43	3.89 ± 0.19	3.21 ± 0.91^a^	3.27 ± 0.40^a^	3.18 ± 0.33	168	3.34 ± 0.19	7.99 ± 0.49	8.90 ± 0.56	3.23 ± 0.37
48	7.42 ± 0.26	12.09 ± 0.53	12.39 ± 0.60	5.00 ± 0.27	197	8.39 ± 0.52	20.76 ± 0.90	22.72 ± 0.59	8.60 ± 0.65
50	5.78 ± 0.32	11.73 ± 0.21	11.00 ± 0.52	4.92 ± 0.49	211	11.02 ± 0.89	26.84 ± 0.52	28.82 ± 0.15	11.05 ± 0.38
57	6.83 ± 0.25	10.91 ± 0.63	12.04 ± 0.28	5.60 ± 0.51	Cont −Hg	1.25 ± 0.02	3.25 ± 0.03^a^	3.19 ± 0.00^a^	1.20 ± 0.01^a^
69I	3.04 ± 0.16	3.41 ± 1.05^a^	4.02 ± 0.21^a^	3.03 ± 0.40	Cont. +Hg	0.04 ± 0.01	0.19 ± 0.00	0.04 ± 0.00	0.00 ± 0.01

*Data with superscripts indicate that there are no significant differences with control without Hg (Duncan’s test). The absence of superscripts means that the means are statistically different from the control without Hg. The shaded cells indicate that the differences with the control without Hg are not statistically significant (p > 0.05).*

Principal component analysis allows ordering the variables according to their variance. The variable that most contributes to the explanation of the model is the root length (63.95% of the variance), followed by the root weight (28.17% of the variance), explaining between them 92.12% of the model. The correlation matrix (Table 4), furthermore, establishes a strong positive correlation between them (*R* = 0.750).

**TABLE 4:** A correlation matrix exists between variables.

	Length (cm)	Weight (g)	CAT	SOD	APX	GR
Length (cm)	1.00					
Weight (g)	0.75	1.00				
CAT	−0.06	0.31	1.00			
SOD	−0.03	0.42	0.90	1.00		
APX	0.06	0.37	0.89	0.97	1.00	
GR	−0.11	0.26	0.93	0.82	0.84	1.00

*Determinant 8.31 × 10^–5^. Enzymatic activity (enzyme mg Prot^–1^ min^–1^). CAT, catalase; SOD, superoxide dismutase; APX, ascorbate peroxidase; and GR, glutathione reductase.*

**FIGURE 2:**
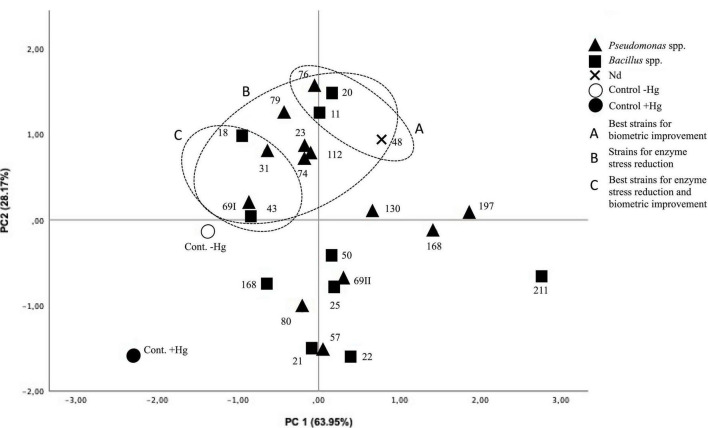
Projection of the two main components (PC1 63.95% and PC2 28.17%). Treatments with higher elongations and root weights segregate toward the quadrant (*x*, *y*). Treatments with a high antioxidant response, which distances them from control in the absence of Hg, segregate toward the (*x*, –*y*) quadrant. Treatments close to control without Hg are grouped in the (–*x*, –*y*) quadrant.

In Figure 2, the two main components (PC1 and PC2) have been projected. The analysis allows the segregation of the strains into two large groups: (i) those that promote biometric improvements in the plant under Hg stress conditions (strains 11, 18, 20, 23, 31, 43, 48, 69I, 74, 76, 79, and 112). Of these, strains 11, 20, 48, and 76 are especially noteworthy. (ii) Those that buffer the antioxidant enzymatic response under Hg stress conditions to levels not significantly different from control in the absence of Hg (strains 18, 31, 43, and 69I).

## Discussion

Exposure of *L. albus* seedlings to increasing concentrations of Hg in the absence of PGPB inocula caused physically detectable phytotoxic effects from 24 h after exposure. Since Hg is a non-essential element in biological systems, there are no specific pathways for its metabolism and/or excretion (Kumari et al., 2020). As Hg accumulates and as observed in our results, the toxic effects become more evident as its concentration increases (Figure 1). In the present study, the phytotoxic effects were evaluated at 120 h of growth. The biometric damages (weight and root length) became significantly notable (*p* < 0.05) from a concentration of 0.5 μg ml^–1^ of HgCl_2_, compared with control without Hg. From this concentration, the radicles appeared thickened and necrotic, something that coincides with what was observed by other authors, working with several herbaceous species, such as *Medicago sativa* (Ortega-Villasante et al., 2007; Zhou et al., 2007) or *Allium sativum* (Zhao et al., 2013). At the highest concentrations (8 and 16 μg ml^–1^), the inhibition of root elongation was total, resulting in weights and lengths close to zero (Figure 1). Given that the objective of the present study was to verify whether PGPB inocula allowed to mitigate, or even reverse, the phytotoxic effects of high Hg pressure exposure, the maximum concentration at which the seeds survived (16 μg ml^–1^) was selected for all further trials. At this concentration, growth inhibition of 33.34% was recorded, results analogous to those observed by Pokorska-Niewiada et al. (2018) in *Secale cereale* (34.6%) at a concentration somewhat higher than that tested in the present work (20 μg ml^–1^).

Recent research focuses on the role that microorganisms that inhabit the rhizosphere can play to help the plant in its development in soils contaminated with Hg (Tiodar et al., 2021). Specifically, there is consensus on the importance of understanding the complex plant-microorganism interactions under stress conditions. In the present study, we proceeded with PGPB inocula isolated from the rhizosphere of plants that naturally grow in the mining district of Almadén (Spain) (Table 1). We agree with other authors that, in a situation of Hg stress, plants are more dependent on microbial activity, and are capable of reducing the harmful effect of metals (Amari et al., 2017). This is what we observed after inoculation with the selected PGPB strains. In the presence of Hg, they reversed the process and produced improvements in root length and weight parameters, as well as in the number of secondary roots, in some cases, even significantly compared with control in the absence of Hg. Such is the case of seeds treated with PGPB 18, 31, 43, 69I, 130, and 168, to a greater extent, and strains 20, 23, 74, 76, 197, and 211, to a lesser degree. Although we agree with the observations of Zafar et al. (2015) and Mukhraiya and Bhat (2017), according to which exposure to Hg produces a significant reduction in seed weight, compared with control in the absence of Hg, it is considered that this parameter is not a good indicator of biometric enhancement. The generalized increase in weight in the presence of Hg and PGPB treatment may be due to abnormal thickening due to Hg pressure rather than adequate root development or an increase in the number of secondary roots (Sabreen and Sugiyama, 2008).

One of the mechanisms by which bacteria can facilitate seed development under abiotic stress conditions is through the synthesis of phytohormones. In the present study, those auxin-producing PGPB (5.5–6.0 μg ml^–1^), such as IAA, have been used as a selection criterion. These hormones are produced in the plant stems and transported to the root tips (Tiodar et al., 2021) where, at moderate concentrations, they favor elongation and promote the development of secondary roots, as occurs in plants inoculated with strains 18, 31, 43, 69I, 130, and 168. On the contrary, IAA concentrations ≥6.0 μg ml^–^has an inhibitory effect on root growth and development. In part, this could be related to ethylene synthesis (Glick, 2003), which translates into an almost null secondary roots development when seeds are inoculated with strains 57, 74, 80, and 211. The regulation of ACC concentrations in response to stress conditions is another mechanism by which bacteria exert a beneficial effect on plants subjected to abiotic stress (Saleem et al., 2007). The hydrolysis of ACC by the bacterial enzyme ACC deaminase (ACCd) produces a decrease in ethylene levels, which increases root development (Glick, 2003; Belimov et al., 2009). Strains 11, 20, 48, and 76 produce significantly higher root elongation than the rest of the traits and controls, possibly due to the combined action of blocking the effect of ethylene produced by the plant under Hg stress conditions *via* ACCd and the activation of cell elongation by the moderate production of IAA. In addition, bacterial siderophores counteract the plant’s inability to accumulate enough Fe from the soil, favoring its bioavailability and avoiding a stressful situation that can promote an increase in ethylene concentration. Unlike what happens with other heavy metals, the role of siderophores in relation to Hg chelation is not known, although it is believed that they could improve Hg absorption in the root (Tiodar et al., 2021). Strains 11, 21, 23, 25, 43, 80, 130, and 146 are good producers of siderophores and could be interesting for further field use in bioremediation processes, where Fe is a limiting element. Strains 11 and 20 (*Bacillus toyonensis*), 48 (not described), and 76 (*Pseudomonas corrugata*), capable of favoring plant development by promoting their growth in the presence of high heavy metals pressure, are interesting candidates for further biotechnological uses, in which an increase in root biomass or plant cover is sought.

Another consequence of Hg exposure is the appearance of oxidative stress with the accumulation of reactive oxygen species (ROS) (Çavuşoğlu et al., 2022) and an increase in the antioxidant enzymatic response (Kim et al., 2017). When plants exceed the threshold of tolerance to heavy metals, their metabolism can be altered, cellular homeostasis is compromised, electron transport chains and the functioning of lipids and proteins are altered, DNA damage is produced and activates the antioxidant defense system (Yang et al., 2018). In the present work, we observe how the exposure of *L. albus* to Hg in the absence of PGPB causes the necrosis of the seeds at 24 h of exposure, which translates into an antioxidant activity tending to zero for the four quantified enzymes. This should not be interpreted as a lack of activity *per se*, but rather the death of the seeds in the absence of PGPB treatment that neutralizes Hg toxicity, as occurs in treatments with PGPB 18, 23, 41, and 69I. The molecular mechanisms by which heavy metals induce ROS formation are not well described, although they have been shown to induce oxidative stress (Cargnelutti et al., 2006; Ajitha et al., 2021; Çavuşoğlu et al., 2022). Traditionally, the accumulation of ROS has been considered a negative effect on plants, the prelude to the cell degradation process. However, there is evidence that it can be a nuclear part of the cellular response system (Flores-Cáceres, 2013), being able to act as secondary messengers that regulate growth and development functions (Foyer and Noctor, 2005). Consequently, it is important for the plant to control the concentration of ROS, but not to eliminate them completely (Çavuşoğlu et al., 2022). In this sense, Kim et al. (2017) studied the role of GR in mitigating the toxicity of Hg (and other heavy metals) on the development of *Arabidopsis thaliana*. They concluded that GR has a much stronger binding affinity for Hg than for Cd, Cu, or Zn, suggesting that tight binding of GR to Hg prevents its uptake, leading to a low accumulation of Hg in plant cells, additionally improving their physiology. This fact is observed in the treatment with strain 18.

In accordance with other authors (Sapre et al., 2019), in the present study, it has been shown that seeds are viable at high Hg concentrations only when treated with PGPB. In the assays carried out in this work, the seeds were developed under the same conditions (environmental, model plant species, and Hg concentration), so the unequal enzymatic responses could be explained by the PGPB effect. For further biotechnological uses of PGPB strains in biotechnological processes, those treatments that manage to mitigate the accumulation of ROS in the plant will be interesting. This effect has been observed by other authors after the use of PGPB from the *Bacillus* sp. (Vardharajula et al., 2011; Moreno-Galván et al., 2020) and *Pseudomonas* sp. genera (Dos Santos et al., 2021). In the present work, strains 31 and 69I (*P. corrugata*) and 18 and 43 (*Bacillus toyonensis*) can maintain the SOD and APX enzymatic responses at low-stress levels, without significant differences compared with control in the absence of Hg (*p* < 0.01). Additionally, strain 18 shows a significant reduction to control levels in the absence of Hg in the GR enzyme. This minimizing effect of enzymatic activity in the presence of abiotic stress has been documented by other authors when working with PGPB inocula and other heavy metals, such as Pb (Abdelkrim et al., 2018), Cu (Fatnassi et al., 2015), Zn (Islam et al., 2014) and Cd (Azimychetabi et al., 2021). According to Kasim et al. (2013), a decrease in antioxidant activity in plants subjected to PGPB treatment can be interpreted as a better adaptation to stress conditions, as it plays a phytoprotective role, which protects plants from abiotic stress. Treatment with strains 18, 31, 43, and 69I could be indicated to improve plant physiology in biotechnological processes involved in the recovery of environments contaminated with heavy metals.

## Conclusion

The recovery of Hg-contaminated sites is a topic of current interest. Phytoremediation of Hg-contaminated soils is an emerging strategy. Species, such as *L. albus* have improved tolerance to inhibitory concentrations of Hg when inoculated with PGPB. The symbiotic relationship of some of these PGPB with plants exerts a phytoprotective role that improves their fitness, from biometrics to oxidative stress alleviation. In particular, we find that treating *L. albus* var. Orden Dorado seeds with strains 31 and 69I (*P. corrugata*), 18 and 43 (*Bacillus toyonensis*) in the presence of high Hg concentrations, minimize the antioxidant response of the seed at these levels of treatment in the absence of Hg. This reflects the phytoprotective nature of these PGPB traits, and as a result, they are postulated as good candidates for further bioremediation processes, in which plant physiology improvement is sought. Similarly, treatment of *L. albus* var. Orden Dorado seedlings with PGPB strains 11 and 20 (*B. toyonensis*), 48 (Not described), and 76 (*P. corrugata*) improves plant biometry (root length and weight) due to the unique combination of their PGPB activities. This makes them good candidates for further bioremediation trials where there is a special interest in achieving notable increases in biomass and plant cover. A detailed investigation is needed to understand the molecular mechanisms underlying Hg uptake, accumulation, and sequestration in plants, and the interactions between plants and their associated microorganisms. In this sense, it is also necessary to unravel the molecular mechanisms by which certain PGPB indirectly prevent Hg from exerting a phytoprotective role as described in this work.

## Data Availability Statement

The original contributions presented in this study are included in the article/supplementary material, further inquiries can be directed to the corresponding author/s.

## Author Contributions

MR and PJ: conceptualization. MR, DG, AP, and PJ: methodology. MR: software, writing—original draft preparation, and visualization. PJ: validation and data curation. MR, AP, and PJ: formal analysis and writing—review and editing. MR and DG: investigation. AP and PJ: resources, supervision, and funding acquisition. AP: project administration. All authors read and agreed to the published version of the manuscript.

## Conflict of Interest

The authors declare that the research was conducted in the absence of any commercial or financial relationships that could be construed as a potential conflict of interest.

## Publisher’s Note

All claims expressed in this article are solely those of the authors and do not necessarily represent those of their affiliated organizations, or those of the publisher, the editors and the reviewers. Any product that may be evaluated in this article, or claim that may be made by its manufacturer, is not guaranteed or endorsed by the publisher.
